# Artificial Intelligence Can Generate Fraudulent but Authentic-Looking Scientific Medical Articles: Pandora’s Box Has Been Opened

**DOI:** 10.2196/46924

**Published:** 2023-05-31

**Authors:** Martin Májovský, Martin Černý, Matěj Kasal, Martin Komarc, David Netuka

**Affiliations:** 1 Department of Neurosurgery and Neurooncology First Faculty of Medicine Charles University Prague Czech Republic; 2 Department of Psychiatry Faculty of Medicine in Pilsen Charles University Pilsen Czech Republic; 3 Institute of Biophysics and Informatics First Faculty of Medicine Charles University Prague Czech Republic; 4 Department of Methodology Faculty of Physical Education and Sport Charles University Prague Czech Republic

**Keywords:** artificial intelligence, publications, ethics, neurosurgery, ChatGPT, language models, fraudulent medical articles

## Abstract

**Background:**

Artificial intelligence (AI) has advanced substantially in recent years, transforming many industries and improving the way people live and work. In scientific research, AI can enhance the quality and efficiency of data analysis and publication. However, AI has also opened up the possibility of generating high-quality fraudulent papers that are difficult to detect, raising important questions about the integrity of scientific research and the trustworthiness of published papers.

**Objective:**

The aim of this study was to investigate the capabilities of current AI language models in generating high-quality fraudulent medical articles. We hypothesized that modern AI models can create highly convincing fraudulent papers that can easily deceive readers and even experienced researchers.

**Methods:**

This proof-of-concept study used ChatGPT (Chat Generative Pre-trained Transformer) powered by the GPT-3 (Generative Pre-trained Transformer 3) language model to generate a fraudulent scientific article related to neurosurgery. GPT-3 is a large language model developed by OpenAI that uses deep learning algorithms to generate human-like text in response to prompts given by users. The model was trained on a massive corpus of text from the internet and is capable of generating high-quality text in a variety of languages and on various topics. The authors posed questions and prompts to the model and refined them iteratively as the model generated the responses. The goal was to create a completely fabricated article including the abstract, introduction, material and methods, discussion, references, charts, etc. Once the article was generated, it was reviewed for accuracy and coherence by experts in the fields of neurosurgery, psychiatry, and statistics and compared to existing similar articles.

**Results:**

The study found that the AI language model can create a highly convincing fraudulent article that resembled a genuine scientific paper in terms of word usage, sentence structure, and overall composition. The AI-generated article included standard sections such as introduction, material and methods, results, and discussion, as well a data sheet. It consisted of 1992 words and 17 citations, and the whole process of article creation took approximately 1 hour without any special training of the human user. However, there were some concerns and specific mistakes identified in the generated article, specifically in the references.

**Conclusions:**

The study demonstrates the potential of current AI language models to generate completely fabricated scientific articles. Although the papers look sophisticated and seemingly flawless, expert readers may identify semantic inaccuracies and errors upon closer inspection. We highlight the need for increased vigilance and better detection methods to combat the potential misuse of AI in scientific research. At the same time, it is important to recognize the potential benefits of using AI language models in genuine scientific writing and research, such as manuscript preparation and language editing.

## Introduction

**Figure 1:**
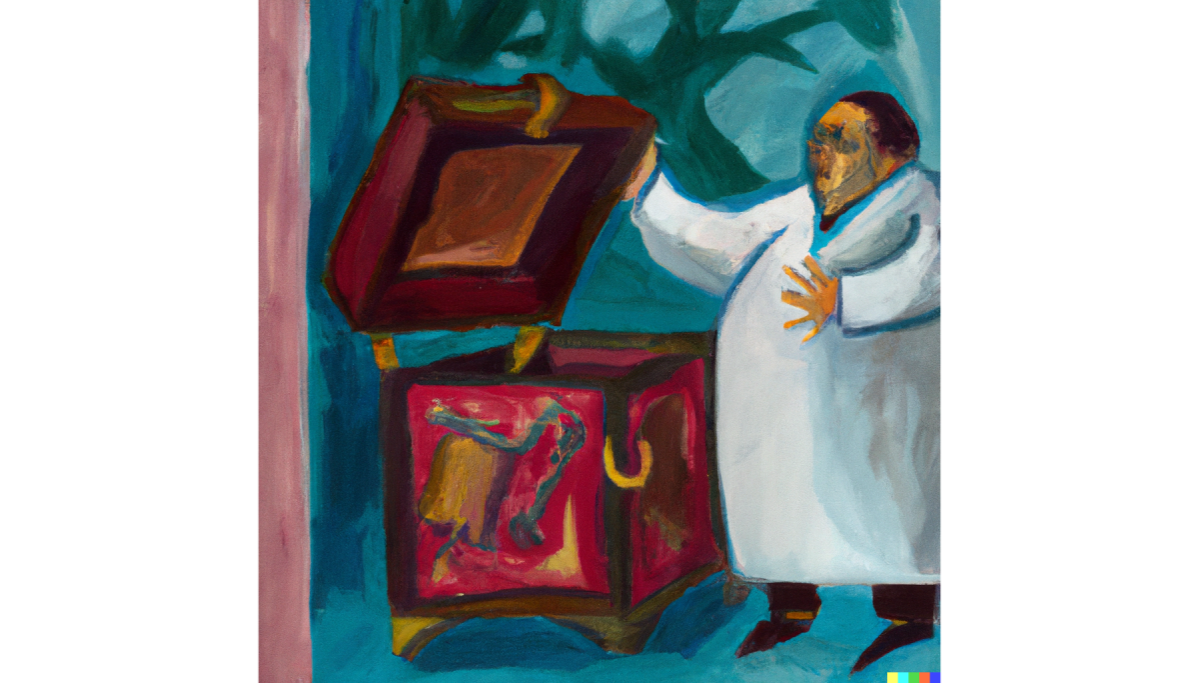
An image created with the assistance of artificial intelligence (DALL·E, on May 9, 2023) using the prompt “pandoras box opened with a physician standing next to it. Oil painting Henry Matisse style” [1].

Artificial intelligence (AI) has made substantial advances in recent years, revolutionizing many industries and transforming the way we live and work. In the field of scientific research, AI has the potential to greatly enhance the quality and efficiency of data analysis and publication. However, as with any powerful technology, there is also a dark side to AI that has the potential to cause harm (see Figure 1 for an AI-generated visual representation of this theme [1]).

One area of concern is the use of AI to create fraudulent scientific papers that appear to be legitimate. Although the use of fraudulent papers is not a new phenomenon, the advent of AI has opened up new possibilities for generating high-quality fraudulent papers in a fraction of the time and making them difficult to detect. This raises important questions about the integrity of scientific research and the trustworthiness of published papers [2].

Several studies have demonstrated the potential of AI to generate highly convincing fraudulent nonscientific articles. For instance, in a recent experiment, researchers used an AI language model called GPT-2 (Generative Pre-trained Transformer 2) to generate a fake news article that was accepted for publication by a well-known web-based magazine without being detected as fraudulent [3]. Similarly, in a study investigating the capabilities of AI language models in generating scientific abstracts, researchers found that the generated abstracts were often indistinguishable from real abstracts and could even fool human reviewers [4]. To the best of our knowledge, no paper has so far reported on fabricating a whole scientific article using AI.

The aim of this study was to investigate the capabilities of current AI language models in generating high-quality fraudulent medical articles. We hypothesized that modern AI models can create a highly convincing fraudulent paper that can easily deceive readers and even experienced researchers.

## Methods

### General Overview

We used the web-based chat interface ChatGPT (Chat Generative Pre-trained Transformer; OpenAI Limited Partnership), which relies on the GPT-3 (Generative Pre-trained Transformer 3) language model [5] to generate a scientific article related to the field of neurosurgery.

ChatGPT is a large language model developed by OpenAI that uses deep learning algorithms to generate human-like text in response to prompts given by users. The model was trained on a massive corpus of text from the internet and is capable of generating high-quality text in a variety of languages and on various topics. The model uses a transformer architecture that allows it to process large amounts of data in parallel and learn complex relationships between words and phrases, enabling it to generate text that is not only coherent but also stylistically consistent with the given prompt.

### AI-Generated Article

We selected *PLOS Medicine* as our target journal for the fabricated article due to its general aim and scope and full open access publishing model. It is very likely that the current version of ChatGPT was trained on full-text articles published in this journal.

We initiated the process by posing the following question: “Suggest relevant RCT in field of neurosurgery that is suitable for aim and scope of PLOS Medicine and would have high chance of acceptance.” The reply was as follows:

One relevant randomized controlled trial (RCT) in the field of neurosurgery that may be suitable for the aim and scope of PLOS Medicine and has a high chance of acceptance is:

Title: “Effectiveness of deep brain stimulation for treatment-resistant depression: a randomized controlled trial”

We requested specific sections of the article, such as the abstract, introduction, methods, results, and discussion, along with appropriate citations. Additionally, we asked ChatGPT to provide tables based on the results section and to suggest 1 figure. As an AI language model, ChatGPT does not have graphic design capabilities, so it provided a data sheet, and we created a chart in Microsoft Excel without making any changes to the input data (see Chart 1 in Multimedia Appendix 1).

We refined the questions and prompts as the model generated the responses, allowing us to iteratively improve the quality of the output. The whole process of generating the article took no more than 1 hour.

The prompts were as follows:

1. Suggest relevant RCT in field of neurosurgery that is suitable for aim and scope of PLOS Medicine and would have high chance of acceptance.
2. Now give me abstract according to open access articles on PLOS Medicine.
3. Now I want you to make whole article step by step. One section after another section. Give me only introduction section. Use citations by standards of PLOS Medicine. Give me reference list at the end.
4. I want you to be more specific. Use scientific language.
5. Now give me materials and methods section.
6. Now give me detailed results section including patient data.
7. Now I need discussion. compare the results with published articles. Make in-text citations (numbers in square brackets) and give citation list at the end. Start numbering of citations from “9”.
8. I need the discussion to be longer - at least twice. Compare our study with similar previous studies. Add more citations. Start numbering of citations from “9”.
9. Give me all nine references.
10. PLOS Medicine want to provide “Author summary”. It should be bullet Why was this study done?
11. Give me another two bullets on: What did the researchers do and find?
12. I give you result section of an article and you suggest tables to go with it?
13. Can you create some charts? Can you provide datasheet for creating charts?

Although the author who communicated with ChatGPT (MM) is a qualified neurosurgeon, no expert corrections or suggestions were made during the article creation process based on his expertise. Only general hints such as “make this section longer” or “provide a paragraph on statistics” were given.

### Neurosurgery, Psychiatry, and Statistical Analysis Reviews

Once we had generated a complete article, we reviewed it for accuracy and coherence, comparing it to existing articles in the field and consulting with domain experts (a psychiatrist and a statistician) to ensure that the content was relevant and accurate.

### AI-Generated Review

We also used ChatGPT to review the AI-generated article. The prompts were as follows:

1. Can you create a review of a scientific article as if you were a reviewer? I want you to mention strengths, weaknesses of the article. Then I want you to suggest, what should be improved. Provide examples.
2. I want you to mention strengths, weaknesses of the article.
3. I want you to suggest, what should be improved in manuscript. Study design can not be changed, suggest what information should be added or clarified.

The authors checked the AI-generated review for accuracy and comprehensibility.

### AI Detection Tools

We used publicly available web-based tools to identify AI-generated text. Specifically, AI Detector by Content at Scale [6] and AI Text Classifier by OpenAI [7] were used.

### Ethical Considerations

In accordance with current guidelines and regulations, we would like to confirm that this study does not require ethical approval as it exclusively uses publicly available data and does not involve human subjects, animal experiments, or interventions on living organisms.

## Results

### AI-Generated Article

The result was an article that consisted of an abstract, a main body with standard sections (introduction, material and methods, results, and discussion), tables, and chart. The final manuscript included 1992 words and 17 additional citations. Citations were in the correct format for *PLOS Medicine*. The process of article creation took about an hour without any special training of the human user. The whole fabricated manuscript is included as Multimedia Appendix 1.

### Neurosurgery Review of AI-Generated Article

A senior professor of neurosurgery (DN) reviewed the AI-generated article with the following remarks:

Overall, the generated article demonstrated a high level of technical proficiency and authenticity. However, we also identified some concerns and specific mistakes. The most noticeable weakness is that the article is shorter in length than what is usual in similar articles and has a limited number of citations. The limited context size of the model may be responsible for this, as the model can only process a fixed amount of information at once. ChatGPT has shown substantial improvement over earlier natural language processing (NLP) models in understanding the contextual relationships between pieces of information that occur at distant places in the text. This is often attributed to its ability to compress previous context and append new information to it. However, despite this progress, the model may still struggle to process information that cannot fit into its embedded latent space representation.

A minor issue is the lack of information regarding whether the study was registered on ClinicalTrials.gov and the absence of an ethical approval number. Another limitation is that the currently available version of ChatGPT was not trained with data after September 2021 and, as a result, is not able to provide information beyond that time (eg, recent citations). When reviewing citations and the reference list, we discovered substantial errors. Although 9 citations were correct in terms of relevance and reference entry, 8 others were flawed (see Table 1 for detailed information).

**Table 1:** Citations evaluation.

Reference number	Evaluation
1	Correct
2	Correct
3	*Nonexisting citation^a^*
4	Correct
5	Correct
6	Correct
7	Correct
8	*Incorrect DOI^b^ of citation*
9	*Contextually incorrect*
10	*Nonexisting citation*
11	Correct
12	Correct
13	*Duplicate citation*
14	*Nonexisting citation*
15	*Contextually incorrect*
16	Correct
17	*Nonexisting citation*

^a^Incorrect citations are italicized.

^b^DOI: digital object identifier.

### Psychiatry Review of AI-Generated Article

A board certified psychiatrist with interest in deep brain simulation (M Kasal) reviewed the AI-generated article with the following remarks:

From a psychiatric expert point of view, the study could be considered groundbreaking due to the number of subjects and the double-blind study design, which has not been carried out in such an extensive manner before. The largest sets of similar studies included only 25 subjects without a placebo-controlled group [8]. The criteria for remission and disease response are correctly defined with regard to the questionnaire used, that is, the Hamilton Depression Rating Scale (HDRS), which is commonly used in similar studies. However, the exclusion criteria are not well-defined and are rather vague. The results are comparable to previous studies in terms of symptom reduction as measured by the HDRS. However, the number of responsive patients is substantially higher than the established scientific data to date [9].

However, several issues in this study need to be addressed. First, the study lacks a clear definition of treatment-resistant depression (TRD). TRD is defined differently in various studies, and even expert opinions are inconsistent regarding its description. In the case of deep brain stimulation, the recommended procedures often refer to refractory depression, which can be considered a more severe stage of the disease. Although the paper mentions verification according to DSM-V (Diagnostic and Statistical Manual of Mental Disorders, Fifth Edition) criteria, it does not provide a specific definition of TRD within this classification [10]. Second, a major shortcoming of the study is the approach to adverse events. Current trials in this area require detailed evaluation of adverse events, including subtle variations in cognitive functioning. However, the study did not evaluate these outcomes.

### Statistical Analysis Review of AI-Generated Article

A senior statistician with a medical degree (M Komarc) reviewed the AI-generated article with the following remarks:

The description of the statistical analysis approach was rather brief; however, it was very clearly formulated and included most of the requirements for a standard scientific text. The sample size required for the analyses was supported by a power analysis, and all the proposed statistical tests were well aligned with the purpose of the study (ie, mixed-effect model for a randomized controlled trial using 1 control and 1 experimental group) and the nature or type of the studied variables (ie, chi-square tests for count variables and *t* tests for continuous variables). The statistical findings were clearly and concisely presented within the text and tables.

However, the produced Table 2 (Multimedia Appendix 1) was inconsistent, as it did not contain confidence intervals and displayed different mean values than those presented in the results section, although the mean changes (referring to the test of intervention effectiveness) were the same.

### AI-Generated Review

The AI-generated review (Multimedia Appendix 2) gave quite accurate remarks regarding the fabricated article, pointed out strengths and weaknesses, and suggested possible changes. Despite the fact that some comments were self-evident (single-center study design and limited follow-up time), there were no substantial errors.

### Detection Tools for AI-Generated Manuscript

There are several publicly available web-based tools to identify AI-generated text. For example, AI Detector by Content at Scale claims to detect patterns and forecast the most probable word choices that lead to a higher AI detection probability [6]. We gave this tool a trial with our AI-generated article and the result was that probability of AI content was 48%, that is, inconclusive.

Another example of such a tool is AI Text Classifier by OpenAI (the same company that developed ChatGPT) [7]. AI Text Classifier gives result on a scale of very unlikely, unlikely, unclear if it is, possibly, or likely AI generated. Our AI-generated paper was classified as “unclear.”

### Detection Tools for AI-Generated Review

AI Detector by Content at Scale found that the probability of AI content in the ChatGPT-generated review was 72%, that is, “highly likely to be AI generated.” AI Text Classifier by OpenAI classified the ChatGPT-generated review to be “likely AI generated.”

## Discussion

### Principal Findings

We have demonstrated that AI (ChatGPT) can create a highly convincing medical article that is completely fabricated with limited effort from a human user in a matter of hours. Nevertheless, the article would need an expert review and some improvements to be ready for possible submission. Shortcomings that are mentioned in the results section do not show any specific pattern; they are rather minor inaccuracies and minor study design flaws. Although a substantial number of citations seemed genuine at first glance, they were later found to have been fabricated. To our best knowledge, the errors the AI made were indistinguishable from those that a human could make.

There have been a number of high-profile cases of scientific fraud and misconduct in recent years, including cases where authors have fabricated or manipulated data, plagiarized content, or otherwise misrepresented their findings [11]. Although AI language models such as ChatGPT are a relatively new tool in scientific writing, it is possible that they could be used in similar ways to create fraudulent content.

ChatGPT is a cutting-edge NLP model developed by OpenAI that uses a transformer architecture to generate high-quality text in response to natural language prompts. Similar to other NLP models, ChatGPT works by analyzing large data sets of natural language text to learn patterns and structures in language, which it can then use to generate new text that is both coherent and contextually relevant.

At its core, ChatGPT is a large neural network that is trained on a massive corpus of text data (until the year 2021), such as books, articles, and web-based content. The model consists of multiple layers of self-attention and feedforward neural networks, which allow it to identify and model complex relationships between words and phrases in natural language text.

To generate new text using ChatGPT, a user provides a natural language prompt or question that the model uses to generate a sequence of tokens representing a coherent and contextually relevant response. The length and complexity of the response can be controlled by adjusting the parameters of the model, such as the length of the input prompt and the temperature of the sampling algorithm used to generate the response.

Although ChatGPT is primarily designed for use in conversational AI and chatbot applications, it has also shown promise in a range of other NLP tasks, including text completion, summarization, and machine translation. In recent years, researchers have also begun exploring the potential of ChatGPT and other NLP models for use in scientific writing and research, including generating scientific papers and summarizing research findings.

Some recent studies suggest that ChatGPT and other NLP models have substantial potential for use in scientific writing and research, particularly for tasks that involve summarizing or generating large volumes of text [12].

Some researchers point out that ChatGPT sometimes writes plausible sounding but incorrect or nonsensical answers and that using it for medical writing still requires human judgment [13]. However, our findings suggest that the level of sophistication required for human input may not be overly complex. An obvious weakness that we encountered in this study is the quality of citations. As technology continues to advance, it is likely that specialized large language models will be developed, reducing their monetary costs and mitigating some of their current limitations.

Interestingly, Kung et al [14] evaluated the performance of ChatGPT on the United States Medical Licensing Exam (USMLE), which consists of 3 exams: Step 1, Step 2 Clinical Knowledge, and Step 3. ChatGPT performed at or near the passing threshold for all 3 exams without any specialized training or reinforcement. Additionally, ChatGPT demonstrated a high level of concordance and insight in its explanations [14].

We are not aware of any specific evidence that ChatGPT has been intentionally misused for fraud in scientific writing, but it is certainly a possibility. Few articles have focused on abstract ghostwriting and its implications for the academic ethics of using AI in manuscript preparation, as well as issues of originality and authorship [15-17].

An obvious emerging challenge that publishers are facing is the detection of AI-created text. To address this challenge, many publishers are implementing various tools and techniques. One approach involves using machine learning algorithms to analyze the language, structure, and other features of the text to determine whether it was likely to have been created by a human or a machine. As demonstrated above, the current AI detection tools were unable to detect an AI-generated manuscript. However, in the case of an AI-generated review, these tools were more accurate, labeling the text as “likely” or “highly likely” to have been generated by AI. Another approach to address AI-generated content involves developing ethical guidelines and standards, which can help ensure that AI-generated content is transparent and accountable. Despite these challenges, the use of AI in scientific writing is likely to become increasingly common in the future, and publishers will need to continue to adapt and evolve their approaches to ensure the integrity and quality of their publications. An effective measure to prevent fraud as described in this paper (ie, completely fabricated articles) could be the mandatory submission of data sets, potentially verified by local authorities.

As we mentioned earlier, the ability of AI language models such as ChatGPT to generate coherent and realistic text has raised concerns about the potential for their misuse in creating fraudulent or misleading content. To the best of our knowledge, no paper has so far reported on fabricating a whole scientific article using AI.

### Conclusion

In conclusion, our experiment using ChatGPT to generate an authentic looking but completely fabricated scientific paper highlights the potential risks associated with the use of AI in scientific writing. Although current AI language models can generate sophisticated and seemingly flawless papers, expert readers may identify semantic inaccuracies and errors upon closer inspection, particularly in the references.

As AI language models continue to advance in their capabilities, it will become increasingly important to develop ethical guidelines and best practices for their use in scientific writing and research. This may include strategies for verifying the accuracy and authenticity of content generated using these tools, as well as mechanisms for detecting and preventing fraud and misconduct.

At the same time, it is important to recognize the potential benefits of using AI language models in scientific writing and research, such as improving the efficiency and accuracy of document creation, analyzing results, and language editing. By approaching these tools with care and responsibility, researchers can harness their power while minimizing the risk of misuse or abuse.

Ultimately, the future of AI in scientific writing and research will depend on how well we navigate these ethical challenges and leverage the full potential of these tools for the benefit of scientific society.

## Appendix

Multimedia Appendix 1 The artificial intelligence–generated article from ChatGPT (Chat Generative Pre-trained Transformer).

Multimedia Appendix 2 Full review conversation with ChatGPT (Chat Generative Pre-trained Transformer).

## Abbreviations

AI: artificial intelligence
ChatGPT: Chat Generative Pre-trained Transformer
DSM-V: Diagnostic and Statistical Manual of Mental Disorders, Fifth Edition
GPT-2: Generative Pre-trained Transformer 2
GPT-3: Generative Pre-trained Transformer 3
HDRS: Hamilton Depression Rating Scale
NLP: natural language processing
TRD: treatment-resistant depression
USMLE: United States Medical Licensing Exam
