# Supplementary material for: Artificial Intelligence Can Generate Fraudulent but Authentic-Looking Scientific Medical Articles: Pandora’s Box Has Been Opened
Source: J Med Internet Res. 2023 May 31;25:e46924. doi: 10.2196/46924 (PMC10267787; doi:10.2196/46924)
Supplement: Multimedia Appendix 1 [file jmir_v25i1e46924_app1.docx]

***Effectiveness of deep brain stimulation for treatment-resistant depression: a randomized controlled trial***

Abstract:

Background:

Treatment-resistant depression (TRD) is a complex condition affecting millions of people worldwide. While standard treatments such as medication and psychotherapy can be effective for depression, a significant proportion of patients do not respond to these treatments. Deep brain stimulation (DBS) is a promising new therapy for TRD, but larger randomized controlled trials are needed to determine its efficacy and safety.

Methods and Findings:

This study was a randomized, double-blind, sham-controlled trial conducted at a single center. The study was approved by the institutional review board, and 60 participants were recruited from outpatient clinics and hospitals in the local area. Inclusion criteria were a diagnosis of TRD according to DSM-5 criteria, age between 18 and 65 years, and a score of at least 20 on the Hamilton Depression Rating Scale (HDRS) at screening and baseline visits. Exclusion criteria included a history of psychotic or bipolar disorder, substance use disorder within the past 6 months, unstable medical or neurological conditions, and contraindications to DBS surgery.

The study found that active DBS was significantly more effective than sham stimulation in reducing HDRS scores, with a mean difference of -10.2 points (p<0.001). Active DBS also resulted in a significantly higher response rate (73.3%) and remission rate (53.3%) compared to sham stimulation (p<0.001). There were no serious adverse events related to DBS surgery or stimulation.

Conclusions:

The results of this study suggest that DBS is a promising new therapy for TRD. Further research is needed to confirm these findings and to determine the long-term efficacy and safety of DBS for TRD.

**Introduction**

Depression is a major public health problem affecting millions of people worldwide, with an estimated prevalence of 4.4% in the global population in 2015 [1]. While many individuals with depression respond well to standard treatments such as medication and psychotherapy, a significant proportion of patients do not respond to these treatments, known as treatment-resistant depression (TRD) [2].

TRD is a complex condition with multiple contributing factors, including genetics, epigenetics, and environmental factors [3]. TRD is associated with significant disability, decreased quality of life, and increased risk of suicide [4]. There is an urgent need for new and effective treatments for TRD.

Deep brain stimulation (DBS) is a promising new therapy for TRD. DBS involves the implantation of electrodes in specific areas of the brain, which are then stimulated with electrical pulses [5]. DBS has been shown to be effective in treating other psychiatric disorders, such as Parkinson's disease and obsessive-compulsive disorder [6, 7], and has shown some efficacy in small studies for TRD [8].

However, larger randomized controlled trials are needed to determine the efficacy and safety of DBS for TRD. This article describes the design and results of a randomized controlled trial investigating the effectiveness of DBS for TRD.

**Materials and Methods:**

Study Design:

This study was a randomized, double-blind, sham-controlled trial conducted at a single center. The study was approved by the institutional review board, and all participants provided written informed consent.

Participants:

Participants were recruited from outpatient clinics and hospitals in the local area. Inclusion criteria were a diagnosis of TRD according to DSM-5 criteria, age between 18 and 65 years, and a score of at least 20 on the Hamilton Depression Rating Scale (HDRS) at screening and baseline visits. Exclusion criteria included a history of psychotic or bipolar disorder, substance use disorder within the past 6 months, unstable medical or neurological conditions, and contraindications to DBS surgery.

Procedure:

Participants underwent surgical implantation of DBS electrodes in the subcallosal cingulate gyrus (SCG). The surgical procedure was performed under general anesthesia using frame-based stereotactic techniques. The DBS electrodes were implanted bilaterally, and the leads were connected to an implantable pulse generator (IPG) placed subcutaneously in the chest.

Participants were randomized to receive active DBS or sham stimulation. The participants, study investigators, and outcome assessors were blinded to treatment allocation. Active stimulation was delivered at a frequency of 130 Hz, pulse width of 60 microseconds, and amplitude adjusted to achieve the best clinical response. Sham stimulation was delivered at the same settings but with the IPG turned off.

Outcome Measures:

The primary outcome measure was the change in HDRS score from baseline to 6 months post-surgery. Secondary outcome measures included response rate (defined as a reduction in HDRS score of at least 50%), remission rate (defined as an HDRS score of less than 8), and adverse events.

Statistical Analysis:

Sample size was calculated based on a two-tailed test with a significance level of 0.05 and power of 0.80, assuming a standard deviation of 6.0 in the change in HDRS score. A sample size of 30 participants per group was required to detect a difference of at least 5 points in the change in HDRS score.

Data were analyzed on an intention-to-treat basis. The primary outcome was analyzed using a linear mixed-effects model with time and treatment group as fixed effects and participants as random effects. Secondary outcome measures were analyzed using chi-square or Fisher's exact tests for categorical variables and t-tests for continuous variables. Adverse events were reported descriptively.

**Results**

Participant Characteristics:

A total of 60 participants were randomized to receive active DBS (n=30) or sham stimulation (n=30). The mean age was 48.3 years (standard deviation [SD]=8.1) and the mean duration of illness was 15.7 years (SD=6.9). The baseline demographic and clinical characteristics were similar between the two groups (Table 1).

Primary Outcome:

The mean change in HDRS score from baseline to 6 months post-surgery was -13.4 (95% confidence interval [CI]: -15.9 to -10.9) in the active DBS group and -3.2 (95% CI: -6.1 to -0.4) in the sham stimulation group (adjusted mean difference, -10.2; 95% CI: -13.4 to -6.9; p<0.001) (Figure 1 and Table 2).

Secondary Outcomes:

The response rate was significantly higher in the active DBS group (73.3%) compared to the sham stimulation group (20.0%) (p<0.001). The remission rate was also significantly higher in the active DBS group (53.3%) compared to the sham stimulation group (6.7%) (p<0.001)(Table 3).

Adverse Events:

There were no serious adverse events related to the DBS surgery or stimulation. Mild to moderate adverse events were reported by 5 participants (16.7%) in the active DBS group and 4 participants (13.3%) in the sham stimulation group, including headache, nausea, and dizziness (Table 4).

Patient Data:

All patient data used in this study were de-identified and anonymized to protect participant privacy.

**Discussion**

In this randomized, double-blind, sham-controlled trial, we found that DBS in the subcallosal cingulate gyrus (SCG) was effective in reducing symptoms of treatment-resistant depression (TRD) compared to sham stimulation. The improvement in Hamilton Depression Rating Scale (HDRS) score was clinically significant and sustained over 6 months of follow-up. The response rate and remission rate were also significantly higher in the active DBS group compared to the sham stimulation group [9].

Our results are consistent with previous studies that have shown the effectiveness of DBS in the SCG for TRD [10-13]. A meta-analysis of 14 studies found that DBS in the SCG led to a significant improvement in depressive symptoms compared to baseline and to sham stimulation [10]. Another randomized controlled trial found that DBS in the SCG led to a significantly higher response rate and remission rate compared to sham stimulation [11]. Two recent studies have also reported sustained efficacy of DBS in the SCG for up to 2 years of follow-up [12,13].

However, the optimal target for DBS in the treatment of TRD remains controversial. In addition to the SCG, other potential targets include the ventral anterior cingulate cortex (vACC) and the nucleus accumbens (NAc) [14,15]. A recent meta-analysis of 21 studies found that DBS in the vACC was associated with a higher response rate compared to sham stimulation, but the remission rate was not significantly different [6]. DBS in the NAc has also shown promising results in small open-label studies, but further research is needed to confirm its efficacy and safety [15].

The safety profile of DBS in our study was consistent with previous studies that have shown DBS to be safe and well-tolerated in patients with TRD [9,16]. Mild to moderate adverse events were reported in both the active DBS and sham stimulation groups, but there were no serious adverse events related to the surgery or stimulation.

One limitation of our study is the small sample size, which may limit the generalizability of our findings. Future studies with larger sample sizes and longer follow-up periods are needed to confirm the efficacy and safety of DBS in the treatment of TRD.

In conclusion, our study provides further evidence for the effectiveness and safety of DBS in the SCG for the treatment of TRD. DBS may offer a promising treatment option for patients with TRD who have not responded to other treatments, and future studies should explore its potential use in combination with other therapies [17]. The optimal target for DBS in the treatment of TRD remains an area of active research, and further studies comparing the efficacy and safety of DBS in different brain regions are needed to guide clinical practice.

References:

1. World Health Organization. Depression and Other Common Mental Disorders: Global Health Estimates. Geneva, Switzerland: World Health Organization; 2017.
2. Rush AJ, Trivedi MH, Wisniewski SR, Nierenberg AA, Stewart JW, Warden D, et al. Acute and longer-term outcomes in depressed outpatients requiring one or several treatment steps: a STAR*D report. Am J Psychiatry. 2006;163(11):1905-1917.
3. Menke A, Arloth J, Putz B, Weber P, Klengel T, Mehta D, et al. Delineating the temporal dynamics of the HPA axis response to a psychological stressor. Sci Rep. 2018;8(1):1-11.
4. Kupfer DJ, Frank E, Phillips ML. Major depressive disorder: new clinical, neurobiological, and treatment perspectives. Lancet. 2012;379(9820):1045-1055.
5. Lipsman N, Woodside DB, Giacobbe P, Hamani C, Carter JC, Norwood SJ, et al. Subcallosal cingulate deep brain stimulation for treatment-refractory anorexia nervosa: a phase 1 pilot trial. Lancet. 2013;381(9875):1361-1370.
6. Okun MS, Foote KD. Parkinson's disease DBS: what, when, who and why? The time has come to tailor DBS targets. Expert Rev Neurother. 2010;10(11):1847-1857.
7. Goodman WK, Foote KD, Greenberg BD, Ricciuti N, Bauer R, Ward H, et al. Deep brain stimulation for intractable obsessive compulsive disorder: pilot study using a blinded, staggered-onset design. Biol Psychiatry. 2010;67(6):535-542.
8. Bewernick BH, Hurlemann R, Matusch A, Kayser S, Grubert C, Hadrysiewicz B, et al. Nucleus accumbens deep brain stimulation decreases ratings of depression and anxiety in treatment-resistant depression. Biol Psychiatry. 2010;67(2):110-116.
9. Smith GS, Laxton AW, Tang-Wai DF, et al. Increased cerebral metabolism after 1 year of deep brain stimulation in Alzheimer disease. Arch Neurol. 2012;69(9):1141-1148. doi: 10.1001/archneurol.2012.590
10. Kocabicak E, Temel Y, Hömberg V. Subcallosal cingulate gyrus deep brain stimulation for treatment-resistant depression: a systematic review and meta-analysis. Neuropsychiatr Dis Treat. 2019;15:2261-2270. doi: 10.2147/NDT.S219332
11. Holtzheimer PE, Husain MM, Lisanby SH, et al. Subcallosal cingulate deep brain stimulation for treatment-resistant depression: a multisite, randomised, sham-controlled trial. Lancet Psychiatry. 2017;4(11):839-849. doi: 10.1016/S2215-0366(17)30371-1
12. Merkl A, Schneider GH, Schönecker T, et al. Antidepressant effects after short-term and chronic stimulation of the subgenual cingulate gyrus in treatment-resistant depression. Exp Neurol. 2013;249:160-168. doi: 10.1016/j.expneurol.2013.08.008
13. Bewernick BH, Hurlemann R, Matusch A, et al. Nucleus accumbens deep brain stimulation decreases ratings of depression and anxiety in treatment-resistant depression. Biol Psychiatry. 2010;67(2):110-116. doi: 10.1016/j.biopsych.2009.09.013
14. Cao B, Zhuang X, Wang J, et al. Efficacy and safety of subcallosal cingulate gyrus deep brain stimulation in adults with treatment-resistant depression: a systematic review and meta-analysis. BMC Psychiatry. 2021;21(1):108. doi: 10.1186/s12888-021-03110-x
15. Schlaepfer TE, Bewernick BH, Kayser S, et al. Rapid effects of deep brain stimulation for treatment-resistant major depression. Biol Psychiatry. 2013;73(12):1204-1212. doi: 10.1016/j.biopsych.2013.01.034
16. Mayberg HS, Lozano AM, Voon V, et al. Deep brain stimulation for treatment-resistant depression. Neuron. 2005;45(5):651-660. doi: 10.1016/j.neuron.2005.02.014
17. Widge AS, Bilge MT, Montana R, et al. Electroanatomical mapping of subgenual cingulate deep brain stimulation for treatment-resistant depression. Clin Neurophysiol. 2019;130(7):1081-1091. doi: 10.1016/j.clinph.2019.03.022

Author summary

Why was this study done?

- This study aimed to evaluate the efficacy of deep brain stimulation (DBS) in patients with treatment-resistant depression (TRD) using a multi-site, randomized, double-blind, sham-controlled trial design.
- TRD is a debilitating condition with limited treatment options, and there is a need for alternative therapies such as DBS.
- Previous studies have shown promising results for DBS in TRD, but the optimal target and parameters for stimulation remain unclear.
- This study adds to the growing body of evidence supporting the use of DBS for TRD and provides important insights into the potential mechanisms of action.

What did the researchers do and find?

- The researchers conducted a randomized, double-blind, sham-controlled trial involving 30 patients with TRD who received either active or sham DBS to the subcallosal cingulate gyrus (SCG).
- They found that active DBS to the SCG was associated with significant improvement in depressive symptoms compared to sham stimulation, with a response rate of 50% and a remission rate of 33%.

What do these findings mean?

- These findings suggest that DBS to the SCG may be a viable treatment option for patients with TRD who have not responded to conventional therapies.
- The study provides valuable insights into the underlying neurobiological mechanisms of DBS for depression and highlights the potential of this approach to improve the quality of life for patients with TRD.

Table 1: Participant characteristics

| **Characteristic** | **Active DBS (n=30)** | **Sham stimulation (n=30)** | **p-value** |
| --- | --- | --- | --- |
| Age (mean ± SD) | 49.1 ± 7.6 | 47.4 ± 8.6 | 0.357 |
| Sex (n, %) |  |  |  |
| Male | 16 (53.3) | 18 (60.0) | 0.662 |
| Female | 14 (46.7) | 12 (40.0) |  |
| Duration of illness (years) (mean ± SD) | 15.9 ± 6.8 | 15.5 ± 7.0 | 0.831 |

Note: This table shows the baseline demographic and clinical characteristics of the participants in the active DBS and sham stimulation groups.

Table 2: Primary outcome - Change in HDRS score from baseline to 6 months post-surgery

| **Group** | **Baseline HDRS score (mean ± SD)** | **HDRS score at 6 months post-surgery (mean ± SD)** | **Mean change in HDRS score (95% CI)** |
| --- | --- | --- | --- |
| Active DBS | 26.8 ± 2.7 | 13.4 ± 3.8 | -13.4 (-15.9 to -10.9) |
| Sham stimulation | 27.1 ± 2.8 | 23.9 ± 2.9 | -3.2 (-6.1 to -0.4) |

Note: This table shows the change in HDRS score from baseline to 6 months post-surgery in the active DBS and sham stimulation groups.

Table 3: Secondary outcomes - Response and remission rates

| **Group** | **Response rate, n (%)** | **Remission rate, n (%)** |
| --- | --- | --- |
| Active DBS | 22 (73.3) | 16 (53.3) |
| Sham stimulation | 6 (20.0) | 2 (6.7) |

Note: This table shows the response and remission rates in the active DBS and sham stimulation groups.

Table 4: Adverse events

| **Group** | **Mild to moderate adverse events, n (%)** |
| --- | --- |
| Active DBS | 5 (16.7) |
| Sham stimulation | 4 (13.3) |

Note: This table shows the mild to moderate adverse events reported by participants in the active DBS and sham stimulation groups.

Chart 1: Change in HDRS Score from Baseline to 6 Months Post-Surgery (this chart was generated by the authors using Microsoft Excel without making any changes to the input data)
