# Supplementary material for: Artificial Intelligence Can Generate Fraudulent but Authentic-Looking Scientific Medical Articles: Pandora’s Box Has Been Opened
Source: J Med Internet Res. 2023 May 31;25:e46924. doi: 10.2196/46924 (PMC10267787; doi:10.2196/46924)
Supplement: Multimedia Appendix 2 [file jmir_v25i1e46924_app2.docx]

**Review by ChatGPT (original version of conversation including prompts)**

***Q: Can you create a review of a scientific article as if you were a reviewer? I want you to mention strengths, weaknesses of the article. Then I want you to suggest, what should be improved. Provide examples.***

*This study was a randomized, double-blind, sham-controlled trial conducted at a single center to investigate the efficacy and safety of deep brain stimulation (DBS) as a therapy for treatment-resistant depression (TRD). The study included 60 participants who were diagnosed with TRD according to DSM-5 criteria, had a score of at least 20 on the Hamilton Depression Rating Scale (HDRS) at screening and baseline visits, and met other inclusion criteria. Exclusion criteria included a history of psychotic or bipolar disorder, substance use disorder within the past 6 months, unstable medical or neurological conditions, and contraindications to DBS surgery.*

*The study found that active DBS was significantly more effective than sham stimulation in reducing HDRS scores, with a mean difference of -10.2 points (p<0.001). Active DBS also resulted in a significantly higher response rate (73.3%) and remission rate (53.3%) compared to sham stimulation (p<0.001). There were no serious adverse events related to DBS surgery or stimulation.*

*The results of this study suggest that DBS is a promising new therapy for TRD, but further research is needed to confirm these findings and to determine the long-term efficacy and safety of DBS for TRD.*

***Q: I want you to mention strengths, weaknesses of the article.***

*Strengths:*

- *The study was conducted as a randomized, double-blind, and sham-controlled trial, which is considered the gold standard in clinical research.*
- *The study had clear inclusion and exclusion criteria, and a standardized assessment tool (HDRS) was used to measure the outcomes.*
- *The study had a sufficient sample size (60 participants) and was conducted at a single center, which reduces the variability in the results.*
- *The study found that DBS was significantly more effective than sham stimulation in reducing HDRS scores, with a high response rate and remission rate.*
- *The study reported no serious adverse events related to DBS surgery or stimulation.*

*Weaknesses:*

- *The study was conducted at a single center, which may limit the generalizability of the results to other settings or populations.*
- *The study had a relatively short follow-up period (6 months), which may not be sufficient to determine the long-term efficacy and safety of DBS for TRD.*
- *The study did not assess the cost-effectiveness of DBS, which is an important consideration for the implementation of this therapy.*
- *The study did not explore the potential mechanisms of action of DBS, which could provide insights into the pathophysiology of TRD and inform the development of new treatments.*
- *The study did not assess the impact of DBS on the quality of life of the participants, which is an important outcome for patients.*

***Q: I want you to suggest, what should be improved in manuscript. Study design can not be changed, suggest what information should be added or clarified***

*Based on the information provided in the article, here are some suggestions for what could be improved or clarified:*

- *Sample characteristics: While the inclusion and exclusion criteria are mentioned, the study does not provide detailed information on the demographic characteristics of the sample. It would be useful to know the age range, gender distribution, and other relevant characteristics of the participants.*
- *Adverse events: While the study mentions that there were no serious adverse events related to DBS surgery or stimulation, it would be helpful to provide more detailed information on the types and frequency of adverse events that were observed. This would help to assess the safety profile of DBS in the context of TRD.*
- *Cost-effectiveness: The study does not provide any information on the cost-effectiveness of DBS compared to standard treatments for TRD. Given the high cost of DBS surgery and the potential benefits of the therapy, it would be important to assess whether the benefits of DBS outweigh its costs.*
- *Generalizability: The study was conducted at a single center, and it is unclear whether the results can be generalized to other settings or populations. It would be useful to conduct larger multi-center trials to assess the generalizability of the findings.*
- *Blinding: The study mentions that it was double-blind, but it does not provide information on how the blinding was achieved or whether the blinding was successful. It would be helpful to provide more information on the blinding procedures used in the study.*
